# Catalytic Oxidation of Propylene, Toluene, Carbon Monoxide, and Carbon Black over Au/CeO_**2**_ Solids: Comparing the Impregnation and the Deposition-Precipitation Methods

**DOI:** 10.1155/2013/824979

**Published:** 2013-09-30

**Authors:** Antoine Aboukaïs, Samer Aouad, Houda El-Ayadi, Mira Skaf, Madona Labaki, Renaud Cousin, Edmond Abi-Aad

**Affiliations:** ^1^Université Lille Nord de France, 59000 Lille, France; ^2^Equipe Catalyse, UCEIV, E.A. 4492, MREI 1, ULCO, 145 avenue Maurice Schumann, 59140 Dunkerque, France; ^3^Department of Chemistry, University of Balamand, P.O. Box 100, Tripoli, Lebanon

## Abstract

Au/CeO_2_ solids were prepared by two methods: deposition-precipitation (DP) and impregnation (Imp). The prepared solids were calcined under air at 400°C. Both types of catalysts have been tested in the total oxidation of propylene, toluene, carbon monoxide, and carbon black. Au/CeO_2_-DP solids were the most reactive owing to the high number of gold nanoparticles and Au^+^ species and the low concentration of Cl^−^ ions present on its surface compared to those observed in Au/CeO_2_-Imp solids.

## 1. Introduction

In recent years, gold-based catalysts receive continuous attention due to their role in various catalytic reactions of commercial and environmental importance. Among these reactions we can quote the oxidation of carbon [1–5] and volatile organic compounds (VOC) [6–11]. The catalytic activity and stability of gold-based compounds depend on the gold particle size, presence of gold cations, metal oxide support, method of preparation, calcination temperature, and pretreatment procedure. However, the preparation method has been reported to directly affect the reactivity of gold-cerium oxide system. The currently employed methods of impregnation, deposition-precipitation, coprecipitation, and chemical vapour deposition strongly influenced the catalytic activity due to the large differences in gold particles size and/or to the availability of active gold sites in close contact with the support defects on the surface [12–14].

Cerium oxide (CeO_2_) has received considerable attention especially in oxidation catalysis [15–20]. This is due to its low temperature reducibility and its oxygen storage and release properties in the presence of noble metal particles. The oxidation/reduction couple (Ce^3+^/Ce^4+^) of ceria particles which are in direct contact with the metal particles promotes the catalytic activity in most cerium-based materials.

The aim of this work is to correlate between the preparation method and the catalytic performance of Au/CeO_2_ catalysts in the total oxidation of propylene (C_3_H_6_), toluene (C_7_H_8_), carbon monoxide (CO), and carbon black (CB).

## 2. Materials and Methods

### 2.1. Preparation of Gold Catalysts

The gold-based catalysts (Au/CeO_2_) were prepared using two different methods: impregnation (Imp) and deposition-precipitation (DP). The used CeO_2_ support was prepared according to [16] and was calcined under air flow (2 L·h^−1^) at 400°C (1°C·min^−1^) for 4 hours.

#### 2.1.1. The Impregnation Method

One gram of the support (CeO_2_) was added to 100 mL of an aqueous solution of tetrachloroauric acid (HAuCl_4_) containing the suitable amount of gold. The solution was left under stirring for two hours before its evaporation at low pressure and 60°C. The obtained solid was dried at 80°C overnight before calcination under air flow (2 L·h^−1^) at 400°C (1°C·min^−1^) for 4 h. The obtained solids are designated *x*Au/CeO_2_-Imp where *x* is the gold weight percent in the calcined solid.

#### 2.1.2. The Deposition-Precipitation Method

One gram of the support (CeO_2_) was added to an aqueous solution of tetrachloroauric acid (HAuCl_4_) at 80°C containing the suitable amount of gold. The pH of the solution was adjusted to 8 by adding NaOH drop by drop under stirring during 4 h. The suspension was filtered and washed several times with hot water in order to eliminate Na^+^ and Cl^−^ ions. The solid was then dried in the oven at 100°C followed by a thermal treatment under air at 400°C (1°C·min^−1^) during 4 h. The obtained solids are designated *x*Au/CeO_2_-DP where *x* is the gold weight percent in the calcined solid.

### 2.2. Catalytic Activity Measurements

The catalytic oxidation tests of gaseous molecules (CO, C_3_H_6_, and C_7_H_8_) were carried out at atmospheric pressure in a continuous flow U-shaped reactor with an internal frit (*ϕ* = 6 mm) on which the catalytic bed is placed. 100 mg of the powder catalyst was loaded in the reactor and then placed in an electrically controlled heating furnace. The reactant gases were injected in the system using mass flow controllers for C_3_H_6_ (6000 ppm) or CO (1000 ppm) and a liquid saturator for C_7_H_8_ (2000 ppm). The total flow was adjusted to 100 N mL·min^−1^ using CO_2_ free industrial air. The product gases were injected into a VARIAN 4900 microgas chromatography allowing the measurement of C_3_H_6_, CO, C_7_H_8_, CO_2_, N_2_, and O_2_ in the stream. Prior to any test, the catalyst (100 mg) was reactivated under air flow (2 L·h^−1^) at 400°C (1°C·min^−1^) during 1 h. The catalytic test towards the combustion of carbon black (CB) (N330 DEGUSSA: specific surface area *S*_sp_ = 76 m^2^·g^−1^, elementary analysis: 97.23 wt.% C; 0.73 wt.% H; 1.16 wt.% O; 0.19 wt.% N; 0.45 wt.% S) was studied by simultaneous thermogravimetric (TG)—differential scanning calorimetry (DSC) analysis with a NETZSCH STA 409 apparatus. Before test, 2 wt.% of CB and 98 wt.% of catalyst were grinded together in an agate mortar for 15 minutes to ensure a tight contact. 50 mg of the mixture was then loaded in an alumina crucible and heated up to 700°C (5°C·min^−1^) under air flow of 75 mL·min^−1^. Each catalytic test was repeated two times for reproducibility checking.

## 3. Results and Discussion

### 3.1. Characterization of Catalysts

The solids obtained using the two different preparation methods have been separately characterized in a previous work [21, 22]. The specific surface areas of the impregnated solids (Imp) were smaller compared to those obtained for the solids prepared by the deposition-precipitation (DP) method. In addition, relatively high contents of chloride anions were present in Imp-solids compared to DP-solids.

Scanning electron microscopy (SEM) and transmission electron microscopy (TEM) showed a heterogeneous distribution of gold particle sizes at the surface of the Imp-solids. In fact, more than 60% of gold was present in the form of large particles (>100 nm) and about only 20% in the form of small nanoparticles (<10 nm) in Imp-solids. On the other hand, only nanoparticles with an average diameter estimated to be equal to 3.9 nm have been detected on the DP-solids. The presence of large particles in the Imp-solids and its absence in the DP-solids were confirmed using the X-ray diffraction technique [21, 22].

The X-ray photoelectron spectroscopy (XPS) showed the presence of Au^0^ and Au^+^ species on both solids types. The Au^+^ species represented only 10% of the total gold atoms on the Imp-solids surface, while its content was about 20% on the DP-solids surface. The small amount of Au^+^ that was still present after calcination under dry air I attributed to gold species located in the proximity of O^2−^ and/or Cl^−^ ions present in the CeO_2_ support. Since elementary analysis showed that the number of Cl present in the DP-solids is negligible compared to that present in the Imp-solids, therefore the ions surrounding the Au^+^ species in both solids types are more likely to be O^2−^ [21, 22].

With the diffuse reflectance ultraviolet/visible spectroscopy (DR-UV/Vis), it was demonstrated, according to the plasmon resonance principle, that gold nanoparticles with diameter smaller than 10 nm are present on both solids types. The intense band obtained for the **4**Au/CeO_2_-DP catalyst indicated that the number of nanoparticles in this solid is greater than that in the impregnated equivalent. In addition, the band obtained at a shorter wavelength demonstrated that the nanoparticles in the DP-solid have spherical shapes and are in weak interaction with the surface of the ceria support [21, 22]. On the contrary, nanoparticles present in the **4**Au/CeO_2_-Imp catalyst can be modelled as hemispheres having more significant interactions with the support surface [21, 22].

The temperature programmed reduction (TPR) results showed that the reduction of Au^+^ into Au^0^ occurs at low temperature on both **4**Au/CeO_2_-DP and **4**Au/CeO_2_-Imp solids with a reduction peak at 94°C and 182°C, respectively. The presence of only gold nanoparticles in the first solid facilitates the reduction of Au^+^ species relatively to those present in **4**Au/CeO_2_-Imp. Consequently, the O^2−^ ions situated near the edge of gold particles in **4**Au/CeO_2_-DP solid are probably more mobile compared to those in **4**Au/CeO_2_-Imp solid [21, 22].

### 3.2. Catalytic Performance of the Solids

#### 3.2.1. Oxidation of Gaseous Probe Molecules

**Figure 1:**
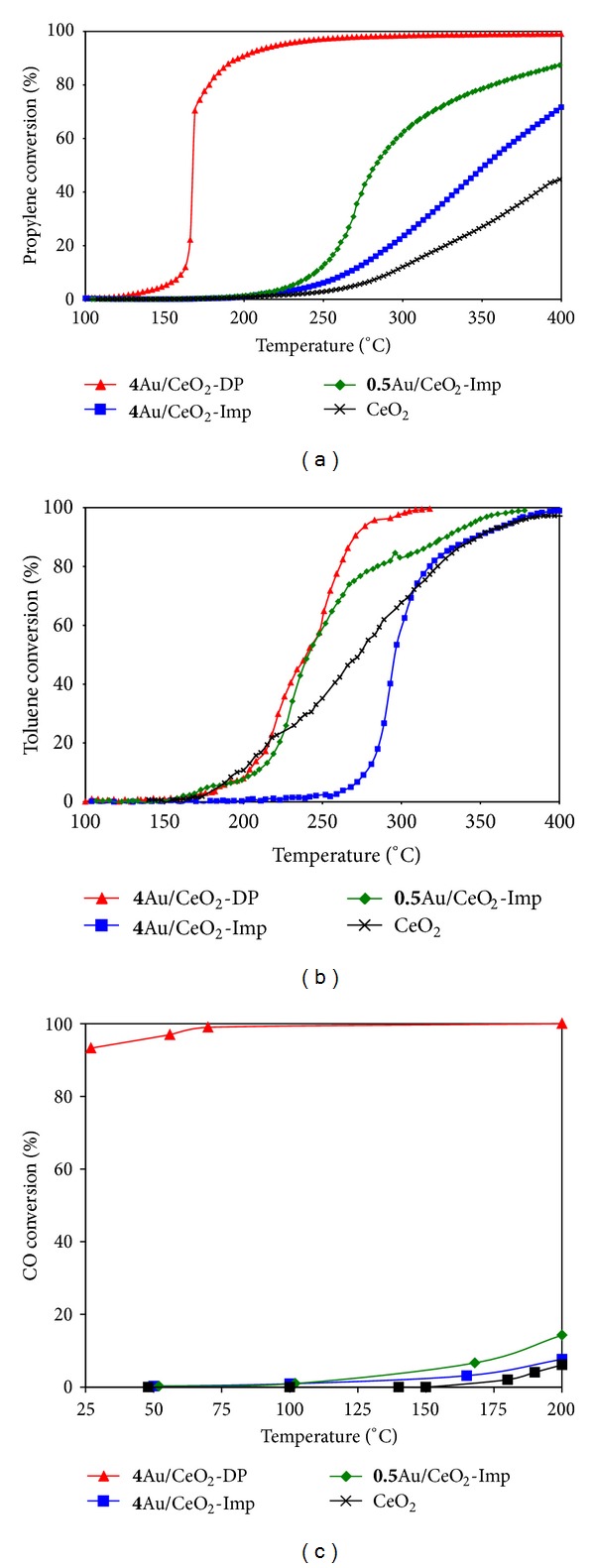
Conversion of (a) propylene, (b) toluene, and (c) CO versus temperature over CeO_2_, **0.5**Au/CeO_2_-Imp, **4**Au/CeO_2_-Imp, and **4**Au/CeO_2_-DP solids.

Figure 1 represents the conversion of propylene, toluene, and carbon monoxide as a function of temperature in the presence of the CeO_2_ support and the gold-based catalysts. Considering propylene oxidation (Figure 1(a)), adding gold to CeO_2_ leads to its conversion at lower temperatures with a selectivity of 100% towards CO_2_ formation.

The **4**Au/CeO_2_-DP catalyst presents the best activity in the considered reaction. The **4**Au/CeO_2_-Imp catalyst is less active which is probably due to its relatively large gold particles compared to those obtained by the DP method. To check the relation between gold particles size and performance in oxidation reactions, a catalyst with 0.5 wt.% gold was prepared using the impregnation method (for low gold contents smaller gold particles are obtained [22]). This latter is more active than the **4**Au/CeO_2_-Imp but remains less active than the **4**Au/CeO_2_-DP catalyst.

Figure 1(b) shows that the impregnated catalyst is less active than the bare support in toluene oxidation with T_50%_ equal to 296°C and 273°C for **4**Au/CeO_2_-Imp and CeO_2_, respectively. Moreover, up to a conversion of 20%, CeO_2_, **0.5**Au/CeO_2_-Imp, and **4**Au/CeO_2_-DP exhibit similar catalytic behaviour. However, CeO_2_ becomes relatively less active for conversions higher than 20%, while **0.5**Au/CeO_2_-Imp and **4**Au/CeO_2_-DP solids remain equally active up to a conversion of 65%. For higher toluene conversions the **4**Au/CeO_2_-DP catalyst becomes the most active among all the tested solids. It is worth to note that in both propylene and toluene oxidation reactions, the only products that evolved were CO_2_ and H_2_O.

Figure 1(c) shows the evolution of the CO conversion as a function of temperature in the presence of the different solids. The support presents a low catalytic activity and the conversion reaches only 6% at 200°C. The conversion is also relatively low for both impregnated catalysts; however, it is slightly higher for **0.5**Au/CeO_2_-Imp especially when the temperature exceeds 125°C. On the other hand, the **4**Au/CeO_2_-DP catalyst was very active with a conversion reaching 93% at room temperature. This high activity has been already related by numerous authors [23–26] to the presence of nanoparticles on the catalyst surface. All the above results confirm that the presence of gold nanoparticles is necessary to oxidize different gaseous molecules at low temperature. In fact, only small nanoparticles are present in the **4**Au/CeO_2_-DP solid, while only 20% of the particles was smaller than 10 nm in the **4**Au/CeO_2_-Imp solid [21, 22]. Moreover, among the impregnated catalysts, the one containing less gold is more active in all reactions. This confirms that the gold particles size is a major determining factor in the catalytic performance of these solids. On the other hand, the high chlorine content resulting from HAuCl_4_ precursor in the impregnated solids may be another reason for its lower performance [2–4]. In addition, numerous authors [27, 28] have related the catalytic activity of Au/CeO_2_ catalysts to the presence of positively charged gold species. It was demonstrated using the XPS technique that the quantity of Au^+^ ions present in the **4**Au/CeO_2_-DP catalyst is two times larger than that present in the **4**Au/CeO_2_-Imp catalyst [21, 22]. This can also explain the better performance of the DP solid.

#### 3.2.2. Oxidation of Carbon Black

**Figure 2:**
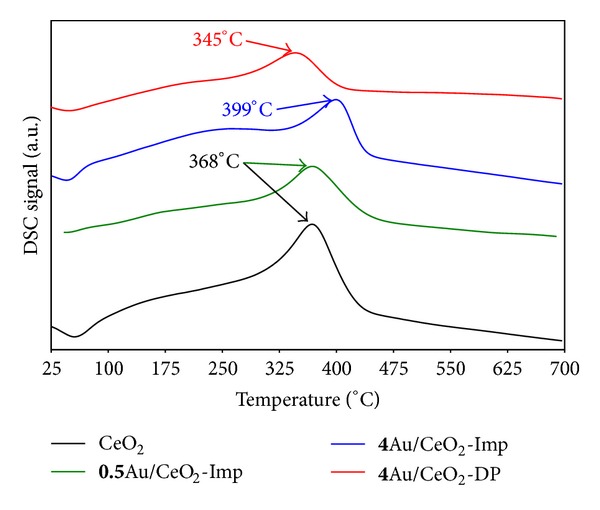
DSC curves obtained during carbon black (2 wt.%) oxidation over CeO_2_, **0.5**Au/CeO_2_-Imp, **4**Au/CeO_2_-Imp, and **4**Au/CeO_2_-DP solids.

Figure 2 shows the DSC curves obtained during carbon black oxidation over the different prepared solids. All TG curves show a 2 wt.% weight loss corresponding to the total elimination of CB present in the mixture (result not shown).

From these latter, two characteristic temperatures can be obtained: *T*_*i*_ (beginning of CB oxidation) and *T*_*f*_ (complete conversion of CB). The maximum of the DSC curve (*T*_max⁡_) corresponds to the temperature at which the reaction rate is the highest. The value Δ*T* = (*T*_*f*_ − *T*_*i*_) is an indication of the reaction rate.  Table 1 lists the different temperatures relevant to all the reactions considered in this paper. The noncatalyzed oxidation of carbon black was done in the presence of SiC as an inert dilution matrix, and the corresponding DSC curve exhibited one exothermic peak with a maximum at 607°C.

Mixing tightly CB to calcined ceria leads to a *T*_max⁡_ = 368°C, a gain of 239°C compared to the noncatalyzed reaction. In fact, due to its oxygen storage capacity (OSC), cerium oxide has been extensively used as a fuel additive, a support for active phases, or even a catalyst in order to accelerate oxidation reaction. The Ce^3+^/Ce^4+^ couple can easily store and release oxygen, making it mobile and readily available to oxidize different types of adsorbed molecules [16, 18, 19]. The addition of gold using the impregnation method leads to a similar reactivity for the low contents (0.5 wt.% of Au) but a decreased reactivity for the **4**Au/CeO_2_-Imp catalyst. This result correlates well with those obtained for gaseous molecules oxidation.

It seems that the high gold content modified the surface characteristics leading to fewer contact points between CB and labile oxygen sites. For the low gold content, the presence of nanoparticles along with some large particles did not affect the catalytic performance. Finally, the presence of **4**Au/CeO_2_-DP catalyst enhanced slightly the catalytic performance compared to ceria with a *T*_max⁡_ = 345°C. The mechanism by which the presence of gold enhances the oxidation of CB is difficult to determine; however, two scenarios may be suggested. First, it has been demonstrated that deposited gold is partly present as Au^+^ species reduce at relatively low temperatures [21]. This is probably a reason for the enhanced reactivity of the **4**Au/CeO_2_-DP catalyst. Second, at moderate temperatures, the vapour pressure of CB increases, and volatile molecules are released. These latter adsorb easily on gold nanoparticles (similar behaviour as probe molecules used in Section 3.2.1) and oxidize in the presence of air with the release of some heat. This phenomenon can be responsible for the initiation of CB oxidation at lower temperature in the presence of **4**Au/CeO_2_-DP catalyst.  Table 1 shows that the CB oxidation reaction is the fastest in the presence of **4**Au/CeO_2_-Imp solid (*ΔT = *72°C) which is expected as the reaction takes place at higher temperatures (*k* = *Ae* ^−*E*_*a*_/*RT*^). However, even if the reaction takes place at lower temperature in the the presence of the **4**Au/CeO_2_-DP catalyst, it is faster (*ΔT* = 110°C) compared to the CeO_2_ catalyzed reaction (*ΔT = *136°C). This result confirms the catalytic activity of the **4**Au/CeO_2_-DP solid. Finally, the catalysts prepared using the impregnation method are less active which is probably due to the presence of large solid particles and chloride ions.

## 4. Conclusion

The catalytic performance of *x*Au/CeO_2_ solids prepared by the deposition-precipitation and the impregnation methods was evaluated in different oxidation reactions. The solid prepared by the deposition-precipitation method was the most active in all the oxidation reactions. This was related to the presence of a higher percentage of reactive Au^+^ species in this solid compared to its percentage in the impregnated solids. The large number of nanoparticles in the **4**Au/CeO_2_-DP catalyst is also a determining factor in its reactivity towards propylene, toluene, carbon monoxide, and carbon black oxidation reactions. It is also shown that the chloride content is another factor that negatively affects the catalytic reactivity. These results can be considered as a proof that the DP solids are more active in the oxidation reactions compared to the Imp solids. However, according to [22], it seems that the Imp solids are more relevant for a different type of reactions that will be detailed in a forthcoming work.

## Figures and Tables

**Table 1:** Characteristic temperatures obtained for the catalyzed propylene, toluene, carbon monoxide, and carbon black oxidation reactions.

Catalysts	*T*_50%_ (°C)		*T*_100%_ (°C)	CB (2 wt.%)	
	C_3_H_6_ (6000 ppm)	C_7_H_8_ (2000 ppm)	CO (1000 ppm)	*T*_max⁡_ (°C)	Δ*T* = *T*_*f*_ − *T*_*i*_ (°C)
CeO_2_	>400	273	>200	368	136
**0.5**Au/CeO_2_-Imp	283	242	>200	368	156
**4**Au/CeO_2_-Imp	352	296	>200	399	72
**4**Au/CeO_2_-DP	167	240	75	345	110
